# Development and application of a fluorescent immunochromatographic assay for metabolite G-DCA quantification in ICP

**DOI:** 10.1016/j.jlr.2026.101039

**Published:** 2026-04-16

**Authors:** Jianyi Gao, Linxia Shuang, Xiaojin Yang, Ruirui Dong, Gaoying Wang, Rong Wang, Ting Zhang

**Affiliations:** 1 Wuxi Maternity and Child Health Care Hospital, Affiliated Women’s Hospital of Jiangnan University, Jiangnan University, Wuxi, China; 2 Wuxi School of Medicine, Jiangnan University, Wuxi, China

**Keywords:** bile acids and salts, competitive assay, dyslipidemias, g-dca, immunochromatography, inflammation, intrahepatic cholestasis of pregnancy, liver, metabolism, pregnancy, small-molecule metabolites

## Abstract

Intrahepatic cholestasis of pregnancy (ICP) is a pregnancy-specific liver disorder characterized by dysregulated bile acid metabolism, which can lead to severe adverse pregnancy outcomes. Glycine-conjugated deoxycholic acid (G-DCA) is a small-molecule metabolite that has been reported to be elevated in the serum of patients with ICP; however, its clinical application remains limited because conventional chromatographic methods for its quantification are time-consuming, costly, and not well-suited for rapid clinical screening. To address this gap, we developed a time-resolved fluorescence immunochromatographic test strip (TRF-ICTS) for the quantitative detection of serum G-DCA. Based on a small-molecule competitive binding design, the assay requires only 10 μl of serum sample and enables rapid detection within 16 min. The TRF-ICTS demonstrated good analytical performance, with a linear range of 0.05–10.0 ng/ml and a detection limit of 0.074 ng/ml, and maintained stable performance over two weeks of storage at both 4°C and 37°C. The results showed good quantitative agreement with LC–MS/MS measurements. Evaluation of sensitivity, specificity, and precision demonstrated that all parameters were within acceptable ranges. Clinical validation using serum samples from ICP patients and healthy pregnant women showed that G-DCA levels were elevated across different trimesters and were more pronounced in patients with severe ICP and adverse pregnancy outcomes. Overall, the developed TRF-ICTS provides a rapid and sensitive method for quantifying G-DCA, which may serve as a complementary biomarker for ICP diagnosis and disease stratification, providing additional insights into bile acid changes following UDCA treatment.

Intrahepatic cholestasis of pregnancy (ICP) is a pregnancy-specific liver disorder that typically occurs during the second and third trimesters (1). Its incidence varies across regions and is relatively high in China, particularly in areas along the Yangtze River basin (2, 3) Clinically, ICP is characterized by persistent pruritus, elevated liver enzymes, and increased circulating total bile acids (TBA), for which ursodeoxycholic acid (UDCA) is commonly used as a first-line treatment (4). However, TBA exhibits limited sensitivity in early-stage ICP and only modest predictive value for adverse perinatal outcomes; therefore, more suitable biomarkers are needed to complement TBA, thereby improving early screening and disease stratification and ultimately enhancing perinatal outcomes (5).

In our previous study, we applied data-independent acquisition (DIA)-based metabolomics to characterize serum metabolic alterations associated with ICP. The results revealed a distinct metabolic shift, with multiple metabolites differentially expressed, including 11 upregulated and 9 downregulated. Among these, glycine-conjugated deoxycholic acid (G-DCA) was significantly elevated in patients with ICP (6). G-DCA is a common conjugated bile acid derived from the conjugation of deoxycholic acid (DCA) with glycine. Accumulation of hydrophobic bile acids, such as DCA, has been shown to disrupt membrane integrity, induce cellular stress, and promote cell injury or apoptosis. Although conjugation with glycine reduces its hydrophobicity, G-DCA may still retain the potential to exert cytotoxic effects (7, 8, 9). Prior studies have shown that in patients with gestational diabetes, G-DCA levels are significantly decreased and negatively correlated with insulin sensitivity (10). In individuals with cystic fibrosis, most bile acid species are elevated, whereas G-DCA does not follow this trend (11). Collectively, these findings highlight the disease-specific alterations of G-DCA, and the marked elevation observed in ICP suggests its potential relevance as a biomarker in this condition.

Liquid chromatography–tandem mass spectrometry (LC–MS/MS) is widely used for the profiling and quantification of serum bile acids, including G-DCA (12, 13). Although this method offers high accuracy, its sample pretreatment is time-consuming and relatively costly, limiting its suitability for rapid clinical screening (14). In contrast, time-resolved fluorescence immunoassay (TRFIA) is a well-established non-radioactive micro-analytical method that utilizes lanthanide chelates (such as europium or samarium) as fluorescent labels and provides ng-level sensitivity, a wide linear range, and cost-effective, user-friendly operation, making it particularly suitable for clinical diagnosis and disease monitoring (15, 16). Although TRFIA has been successfully applied to the detection of various biomacromolecules, including proteins, hormones, and antibodies, its application in the quantitative analysis of serum small-molecule metabolites remains limited (17, 18, 19). In this study, a time-resolved fluorescence immunochromatographic test strip (TRF-ICTS) was developed using an indirect competitive immunoassay format for the rapid and quantitative determination of serum G-DCA levels.

To further validate the feasibility and clinical applicability of the developed TRF-ICTS, systematic optimization and evaluation were performed. First, key assay parameters, including the EDC/NHS-mediated coupling conditions of carboxyl microspheres, MES buffer pH, antibody concentration, and reaction time, were systematically optimized to establish the optimal reaction system. Subsequently, the analytical performance of the test strip, including precision, accuracy, stability, and specificity, was evaluated, and acceptable results were obtained. Finally, the clinical application potential of the test strip was assessed. The TRF-ICTS requires only 10 μl of serum and enables rapid and accurate quantitative detection within 16 min, supporting the potential of G-DCA as a promising biomarker to complement TBA in clinical diagnosis.

## Materials and methods

### Chemicals and reagents

The G-DCA standard (C_26_H_43_NO_5_, purity 97%) was obtained from Best Biological Co., Ltd. The complete antigen G-DCA–OVA and the mouse monoclonal antibody against G-DCA (G-DCA-mAb) were supplied by Beijing Youke Biotechnology Co., Ltd. (Beijing, China). Carboxyl-functionalized Eu^3+^ fluorescent microspheres (Eu^3+^-FM) were obtained from Baina New Material Technology Co., Ltd. (Taizhou, China). DCA was purchased from Shanghai Yuanye Bio-Technology Co., Ltd. Cholic acid (CA), chenodeoxycholic acid (CDCA), ursodeoxycholic acid (UDCA), taurodeoxycholic acid (TDCA), taurochenodeoxycholic acid (TCDCA), and glycochenodeoxycholic acid (GCDCA) were purchased from Aladdin Biochemical Technology Co., Ltd.

### Patients

Participant enrollment followed the 2024 clinical management guidelines for ICP (20), and none of the participants had received UDCA therapy prior to enrollment. From December 2023 to March 2025, serum samples were collected from 54 healthy pregnant women and 61 patients with ICP in the third trimester at Wuxi Maternal and Child Health Hospital. Additionally, serum samples from the first and second trimesters were collected from the same individuals. All samples were collected into 1.5 ml centrifuge tubes and stored at −80°C until analysis. This study was conducted in accordance with the Declaration of Helsinki and was approved by the Institutional Review Board of Wuxi Maternal and Child Health Hospital (Approval No. 2021-916).

### LC-MS/MS

Serum samples were pretreated as previously described with minor modifications (21). 100 μl of serum was mixed with 400 μl of cold methanol containing an internal standard and vortexed for 2 min. The mixture was then ultrasonicated in an ice–water bath for 15 min, followed by centrifugation at 13,000 g for 20 min at 4°C. Subsequently, 400 μl of the supernatant was transferred to a fresh centrifuge tube and evaporated to dryness. The residue was reconstituted in 100 μl of 20% methanol, followed by vortexing, ultrasonication, and centrifugation under the same conditions. Finally, the supernatant was transferred to an autosampler vial for quantitative LC–MS/MS analysis using a QTRAP 5500 liquid chromatography–triple quadrupole system (AB Sciex, Framingham, MA, USA).

### Preparation of Eu^3+^-FM-labeled G-DCA-mAb

In a two-step conjugation procedure, 10 μl of Eu^3+^-labeled carboxyl microspheres was mixed with 800 μl of MES buffer (0.05 M, pH 5.5). After thorough mixing, 5 μl of 1-ethyl-3-(3-dimethylaminopropyl)carbodiimide (EDC, 2 mg/ml) and 5 μl of N-hydroxysuccinimide (NHS, 2 mg/ml) were added. The mixture was vortexed and briefly sonicated (3 min × 3), followed by incubation for 30 min at room temperature in the dark. The activated microspheres were centrifuged (13,000 g, 15 min, 4°C) and washed three times with Tris–HCl buffer (0.05 M, pH 7.2) containing 0.05% Tween-20. Subsequently, 0.5 μg of G-DCA-mAb was added, and the mixture was incubated with gentle shaking at 500 rpm for 3 h. After conjugation, 200 μl of BSA blocking solution was added and incubated for an additional 30 min in the dark. The product was centrifuged (13,000 g, 15 min, 4°C) and washed three times to remove unbound antibodies, then redispersed in 400 μl of preservation buffer and stored at 4°C in the dark.

### Assembly of a time-resolved fluorescence immunochromatographic test strip for G-DCA detection (G-DCA TRF-ICTS)

The test strip consisted of a sample pad, NC membrane, and absorbent pad. G-DCA-OVA and goat anti-mouse IgG were diluted to 1.0 mg/ml in PBS (0.01 M, pH 7.4) containing 5% sucrose and dispensed onto the NC membrane using an XYZ3050 platform at 1.0 μl/cm to form the test (T) and control (C) lines, respectively. The T and C lines were spaced approximately 4 mm apart, and the membrane was dried at 37°C for 3 h. The components were assembled on a PVC backing card with an overlap of ∼2 mm between adjacent components, and the sheet was cut into strips (4 mm × 60 mm). The strips were sealed with desiccant and stored at 4°C until use.

### Usage of the G-DCA TRF-ICTS

A 10 μl serum sample was mixed with 70 μl of Tris–HCl buffer (0.05 M, pH 7.2) containing Eu^3+^-labeled anti-G-DCA antibody-conjugated microspheres, vortexed, and applied to the sample pad, followed by incubation at 37°C for 16 min. The fluorescence signal at the T line was measured using an HG-98 immunofluorescence analyzer (Huguo Scientific Instrument Co., Ltd).

### Performance of the G-DCA TRF-ICTS

#### Standard curve and LOD

G-DCA standards (0.05–10.0 ng/ml) were prepared by serial dilution in Tris–HCl buffer, which served as the negative control. T_0_ and T denote the fluorescence intensities at the T line of the negative and positive samples, respectively. The log concentration of G-DCA was plotted against the T/T_0_ ratio to generate the calibration curve by linear regression. Each concentration was measured in triplicate. For limit of detection (LOD) determination, negative samples were measured ten times, and the mean and standard deviation (SD) of the T/T_0_ ratio were calculated. The LOD was defined as mean −3 × SD (22), reflecting the inverse relationship between signal intensity and G-DCA concentration in the competitive assay, and the corresponding concentration was obtained from the calibration curve.

#### Precision

Eight sets of G-DCA TRF-ICTS were randomly selected from the same batch and from different batches to evaluate inter- and intra-assay variability, respectively. Negative samples and samples at 1.0 ng/ml G-DCA were tested. Each set was measured in triplicate. The fluorescence intensity at the T line was recorded, and results were expressed as T/T_0_ ratios. The coefficient of variation (CV, %) was calculated as CV = SD/mean × 100%.

#### Accuracy and recovery

Serum samples spiked with G-DCA at low (0.1 ng/ml), medium (1.0 ng/ml), and high (10 ng/ml) concentrations were analyzed using the TRF-ICTS. Each concentration was measured in triplicate. The CV was calculated, and the recovery was determined using the formula Recovery (%) = (measured concentration/added concentration) × 100%.

#### Stability

TRF-ICTS from the same production batch were stored at 4°C and 37°C under light-protected conditions for 16 days. Fluorescence signals at the T line were measured at predefined time points (days 1, 2, 4, 6, 8, 10, 12, and 16) using negative controls and samples containing 1.0 ng/ml G-DCA. The results were expressed as T/T_0_ ratios, and the CV was calculated to assess the stability of the assay over time.

#### Specificity and interference evaluation

The specificity of the TRF-ICTS was assessed by evaluating cross-reactivity with structurally related bile acids, including DCA, CA, CDCA, UDCA, TDCA, TCDCA, and GCDCA, each tested at a concentration of 1.50 ng/ml.

Interference was evaluated by adding common endogenous substances to samples containing G-DCA (10 ng/ml), with final concentrations of bilirubin (500 μmol/L), hemoglobin (5 g/L), and triglycerides (15 mmol/L), followed by detection using the TRF-ICTS. All experiments were performed in triplicate. The bias (%) was calculated as: Bias (%) = (C_interference − C_baseline)/C_baseline × 100%, where C_interference and C_baseline represent the measured concentrations in the presence and absence of interference, respectively.

### Statistical analysis

Statistical analyses were performed using GraphPad Prism and SPSS 27.0 (IBM). The Shapiro–Wilk test was used to assess normality. Continuous variables were expressed as mean ± SD or median (IQR). Group comparisons were performed using Student’s *t* test or one-way analysis of variance (ANOVA) for normally distributed data, while paired comparisons were conducted using a paired *t* test or Wilcoxon signed-rank test. Correlations were assessed using Pearson or Spearman’s rank correlation. Receiver operating characteristic (ROC) curves were constructed to evaluate the diagnostic performance of G-DCA, and the area under the curve (AUC) with 95% confidence intervals (CIs) was calculated. The optimal cut-off value was determined using Youden’s index. An AUC > 0.70 was considered acceptable, and *P*< 0.05 was considered statistically significant.

## Results

### Sample clinical data

The clinical characteristics and pregnancy outcomes of women with ICP and normal pregnancies during the third trimester were compared. The ICP group showed higher levels of TBA, total bilirubin (TBIL), direct bilirubin (DBIL), alanine aminotransferase (ALT), and aspartate aminotransferase (AST), along with lower gestational age at delivery and neonatal birth weight, compared with the control group (*P*< 0.05) (Table 1).

**Table1:** Baseline clinical information of the enrolled patients

Variable	ICP (n = 61)	Control (n = 54)	*P*-value
Maternal age	29.0 (27.0, 33.0)	31.0 (27.0, 33.3)	0.75
TBA (μmol/L)	29.3 (23.3, 46.0)	2.1 (1.5, 3.1)	0.000∗∗∗∗
TBIL (μmol/L)	9.0 (7.0, 10.3)	7.3 (6.3, 9.1)	0.02∗
DBIL (μmol/L)	2.3 (1.9, 3.1)	1.9 (1.5, 2.3)	0.000∗∗∗∗
ALT (IU/L)	16.4 (7.1,47.0)	9.3 (7.7,13.0)	0.00∗∗∗
AST (IU/L)	21.0 (15.3,31.7)	15.9 (13.5,20.2)	0.000∗∗∗∗
Gestational weeks (Delivery)	38.6 (36.6, 39.5)	39.5 (39.0, 40.3)	0.000∗∗∗∗
Newborn weight (g)	3,030 (2755, 3,250)	3,470 (3,115, 3,658)	0.000∗∗∗∗

Data are presented as median (25th, 75th percentiles) for variables with non-normal distributions. Statistical significance is indicated by ∗*P*< 0.05, ∗∗∗*P*< 0.001 and ∗∗∗∗*P*< 0.0001.

### Working principle and detection mechanism of the G-DCA TRF-ICTS

**Fig.1:**
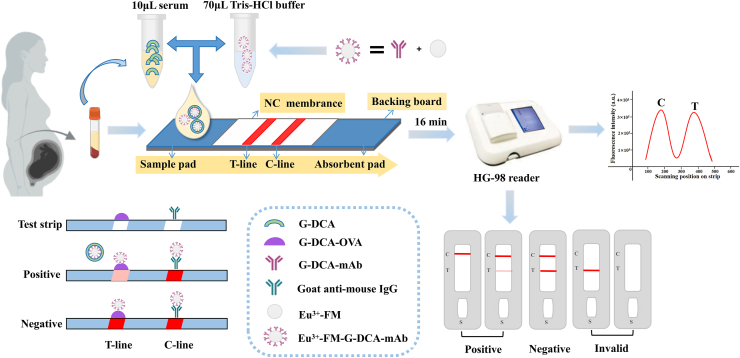
Schematic illustration of the G-DCA TRF-ICTS assay workflow and detection principle.

The G-DCA TRF-ICTS operates based on a competitive immunoassay mechanism (23). As illustrated in Figure 1, the device consists of a sample pad, a NC membrane, and an absorbent pad. The serum sample was mixed with a Tris–HCl buffer containing the Eu^3+^-FM–G-DCA-mAb conjugate and applied to the test strip, allowing migration along the strip via capillary action. After incubation at 37°C for 16 min, the Eu^3+^-FM–G-DCA-mAb conjugate binds to the immobilized antigen on the T line in the absence of G-DCA, generating a strong fluorescent signal detected by the HG-98 immunofluorescence analyzer, while unbound conjugate is captured at the C line by goat anti-mouse IgG. With increasing G-DCA concentration, competition for antibody binding leads to a progressive decrease in fluorescence intensity at the T line, and the signal is inversely proportional to the G-DCA concentration.

### Optimization and analytical performance of the G-DCA TRF-ICTS

**Fig. 2:**
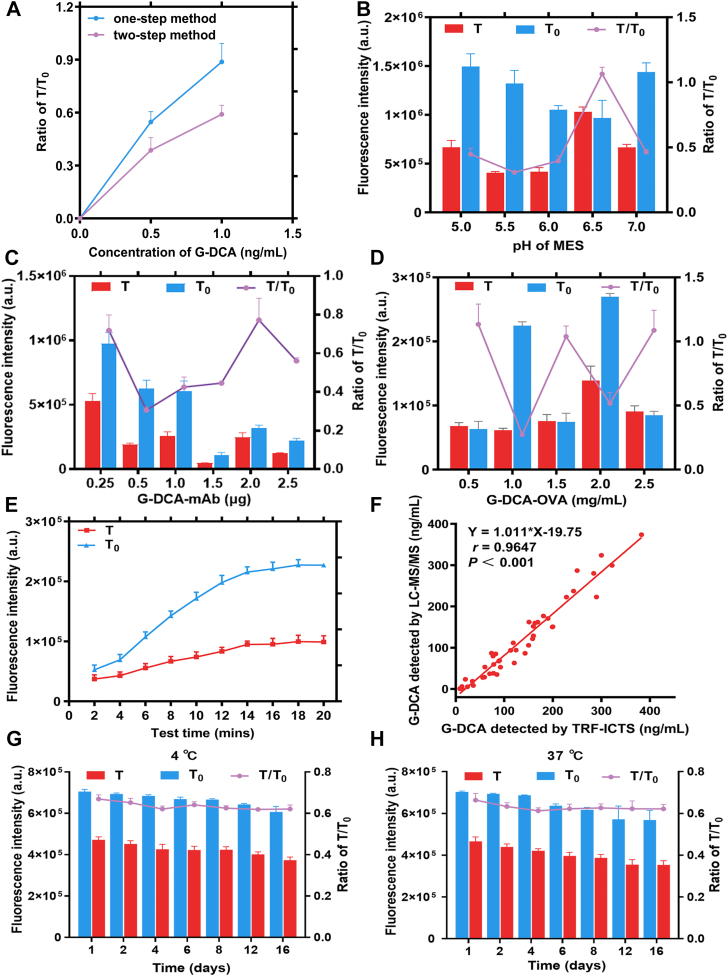
Systematic optimization and comprehensive performance evaluation of the G-DCA TRF-ICTS. A: Optimization of EDC/NHS coupling strategies. B: Optimization of MES buffer pH. C: Optimization of G-DCA-mAb dosage for Eu^3+^-FM coupling. D: Optimization of G-DCA-OVA concentration on the T line. E: Optimization of assay response time. F: Correlation between G-DCA concentrations determined by TRF-ICTS and LC–MS/MS (n = 50). G: Stability of G-DCA TRF-ICTS under light-protected conditions at 4°C. H: Stability of G-DCA TRF-ICTS under light-protected conditions at 37°C. The optimization efficiency was evaluated based on the T/T_0_ ratio, where T represents the fluorescence intensity of positive samples and T_0_ represents that of negative samples. Each condition was tested in triplicate.

As shown in Figure 2A, the two-step conjugation method achieved higher labeling efficiency than the conventional one-step procedure, as reflected by a lower T/T_0_ ratio. Optimal assay performance was achieved at pH 5.5 in MES buffer, with 0.5 μg G-DCA-mAb and a T line coating of 1.0 mg/ml G-DCA-OVA, corresponding to the lowest T/T_0_ value (Fig. 2B–D). The optimal reaction time was 16 min, at which the T/T_0_ signal reached a stable plateau (Fig. 2E). A strong correlation was observed between the TRF-ICTS and LC–MS/MS methods (Fig. 2F; n = 50, r = 0.9647, *P*< 0.001), indicating good agreement between the two methods. Accelerated stability testing demonstrated that the G-DCA TRF-ICTS maintained stable T/T_0_ signals over two weeks at 4°C, with consistent performance also observed under accelerated conditions at 37°C (Fig. 2G, H; Table 2). Detailed optimization procedures are provided in Supplemental Material S1.

**Table 2:** Accelerated stability evaluation of the G-DCA TRF-ICTS

Time (days)	T_0_ (a.u.)	4°C		SD	T_0_ (a.u.)	37°C		SD
		T (a.u.)	T/T_0_ (%)			T (a.u.)	T/T_0_ (%)	
1	716,505	468,421	65.38	2.25	706,000	442,343	62.65	3.23
2	696,995	443,042	63.56	2.06	691,152	435,562	63.02	2.32
4	687,542	427,427	62.17	3.01	688,993	429,500	62.34	1.45
6	675,571	435,293	64.43	3.69	645,343	416,125	64.48	2.21
8	662,003	407,296	61.52	1.91	630,531	404,312	64.12	1.91
12	646,807	394,100	60.93	1.83	622,031	380,888	61.23	3.80
16	637,531	385,482	60.46	2.31	612,062	375,824	61.40	2.12
Mean	674,708	423,009	62.64		656,587	412,079	62.75	
SD	28,081	29,226	1.86		38,010	26,252	1.24	
CV (%)	4.16	6.91	2.96		5.79	6.37	1.98	

T_0_ and T denote the fluorescence signals of the negative control and 1.0 ng/ml G-DCA samples, respectively. T/T_0_ (%) was used to normalize variation and assess assay stability. Measurements were performed in triplicate at each time point.

### Method validation of the G-DCA TRF-ICTS

**Fig. 3:**
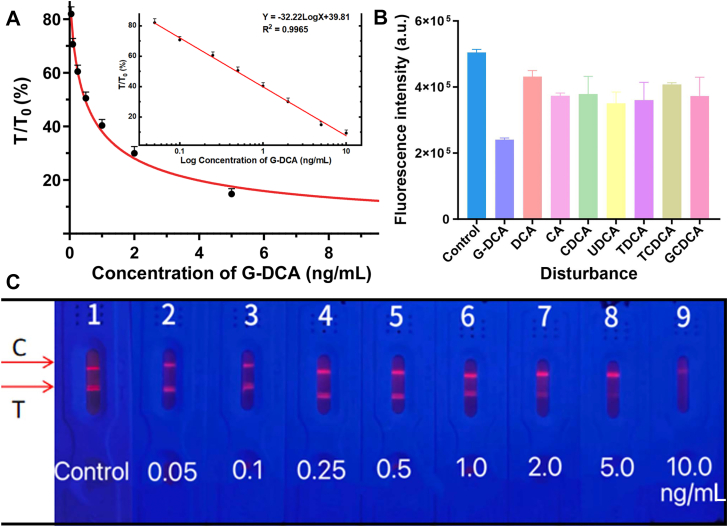
Calibration and analytical performance of the G-DCA TRF-ICTS. A: Calibration curve of G-DCA based on the T/T_0_ ratio. The inset presents the linear regression of T/T_0_ versus the logarithm of G-DCA concentration, including the fitted regression equation (Y = −32.22 logX + 39.81) and correlation coefficient (*R*^2^= 0.9965). B: Specificity evaluation of the G-DCA TRF-ICTS against structurally related bile acids (including DCA, CA, CDCA, UDCA, TDCA, TCDCA, and GCDCA). C: Representative fluorescence images of the TRF-ICTS at different G-DCA concentrations under 365 nm UV illumination. The fluorescence intensity of the T line decreased with increasing G-DCA concentration, consistent with the competitive immunoassay principle. Each condition was tested in triplicate).

The developed G-DCA TRF-ICTS showed a LOD of 0.074 ng/ml and a linear range of 0.05–10.0 ng/ml (Y = −32.22 log X + 39.81, *R*^2^= 0.9965) (Fig. 3A). Samples exceeding the upper limit were quantified after appropriate dilution. Under 365 nm UV illumination, a clear red fluorescent signal was observed at the T line, showing a concentration-dependent decrease with increasing G-DCA levels. (Fig. 3C). The assay exhibited specificity toward G-DCA, with structurally related bile acids showing no significant interference (Fig. 3B). Inter- and intra-assay CVs were 2.68% and 1.22%, respectively (Table 3). Interference from common serum interferents, including bilirubin (500 μmol/L), hemoglobin (5 g/L), and triglycerides (15 mmol/L), had minimal effects on the assay. As shown in Table 4, the corresponding bias values were 7.09%, 5.53%, and 2.51%, respectively, all of which are within the acceptable range of <10%. The recovery of TRF-ICTS ranged from 99.89% to 101.33%, comparable to LC-MS/MS (97.67%–101.17%). The CV values were 2.23%–6.57%, slightly higher than LC-MS/MS (1.77%–3.13%) but within acceptable limits (Table 5).

**Table 3:** Precision evaluation of the G-DCA TRF-ICTS

Inter-assay	T (a.u.)	T_0_ (a.u.)	T/T_0_ (%)	Intra-assay	T (a.u.)	T_0_ (a.u.)	T/T_0_ (%)
1	315,671	403,375	78.26	1	318,317	410,468	77.55
2	299,906	374,312	80.12	2	299,958	383,578	78.20
3	340,781	425,937	80.00	3	315,187	404,156	77.99
4	293,218	363,343	80.70	4	295,828	391,963	75.47
5	294,625	380,062	77.52	5	296,453	385,583	76.88
6	306,500	397,250	77.16	6	311,573	397,489	78.39
7	304,984	408,778	74.61	7	320,650	412,036	77.82
8	304,781	398,718	76.44	8	316,849	406,296	77.98
Mean	307,558	393,972	78.10	Mean	309,352	398,946	77.54
SD	15,196	20,287	2.09	SD	10,287	11,025	0.95
CV (%)	4.94	5.15	2.68	CV (%)	3.33	2.76	1.23

Eight replicate measurements using test strips from the same batch were performed to evaluate intra-assay precision, whereas test strips from eight different batches were measured on different days to assess inter-assay precision. Each sample was measured in triplicate.

**Table 4:** Evaluation of interference effects on the G-DCA TRF-ICTS

Substance	Concentration	Bias (%)
Bilirubin	500 μmol/L	7.09
Hemoglobin	5 g/L	5.53
Triglycerides	15 mmol/L	2.51

Bias (%) was calculated by comparing the measured concentration of 10 ng/ml G-DCA with and without the addition of interfering substances. Each sample was measured in triplicate. A bias of <10% was considered acceptable.

**Table 5:** Comparison of recovery and precision of G-DCA TRF-ICTS and LC-MS/MS

Added Concentration (ng/ml)	G-DCA TRF-ICTS			LC-MS/MS		
	Measured Concentration (ng/ml)	Recovery Rate (%)	CV (%)	Measured Concentration (ng/ml)	Recovery Rate (%)	CV (%)
0.1	0.101 ± 0.007	101.33	6.57	0.098 ± 0.003	97.67	3.13
1.0	1.004 ± 0.046	100.37	4.55	1.012 ± 0.026	101.17	2.55
10	9.989 ± 0.222	99.89	2.23	10.052 ± 0.178	100.52	1.77

Recovery and precision were evaluated at three spiked concentrations (0.1, 1.0, and 10 ng/ml). Measured concentrations are presented as mean ± SD, with each concentration measured in triplicate.

### Diagnostic performance and clinical correlation of serum G-DCA in ICP

**Fig. 4:**
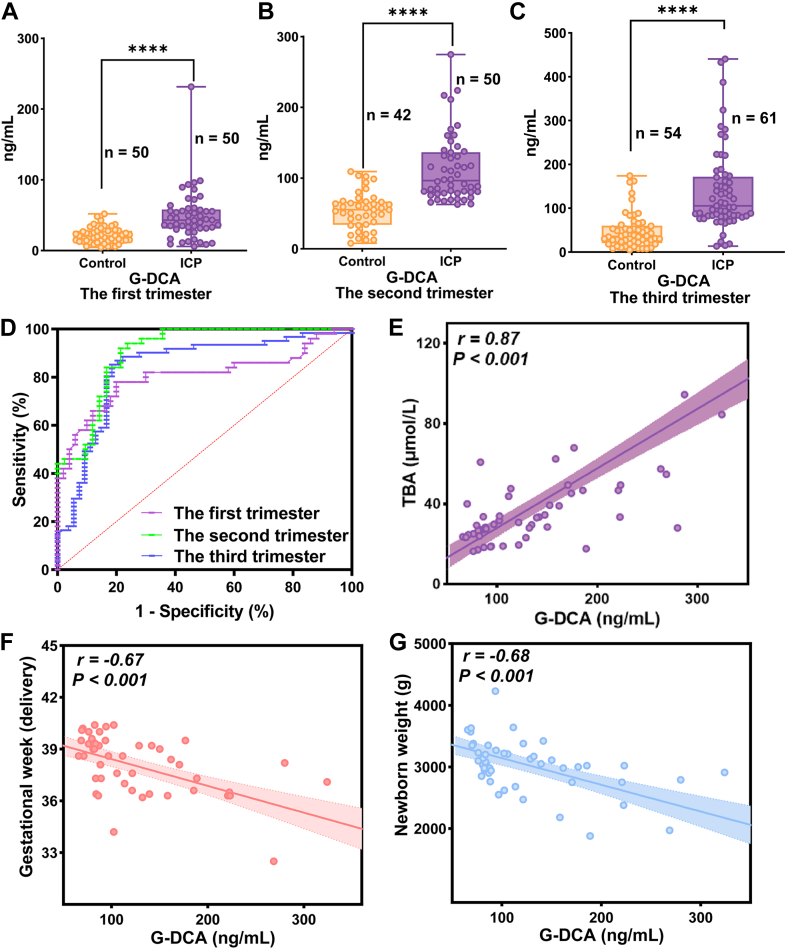
Serum G-DCA levels and diagnostic performance in ICP. A–C: Serum G-DCA concentrations in healthy controls and ICP patients during the first (n = 50 vs. 50), second (n = 42 vs. 50), and third trimesters (n = 54 vs. 61), respectively. Significantly higher levels were observed in ICP patients compared with controls (*P*< 0.0001). D: ROC curves of G-DCA for ICP diagnosis in different trimesters, with AUC values of 0.805, 0.906, and 0.864 for the first, second, and third trimesters, respectively. E–G: Correlation analyses in the third trimester showing a positive correlation between G-DCA and TBA (r = 0.87, *P*< 0.001), and negative correlations with gestational age at delivery (r = −0.67, *P*< 0.001) and neonatal birth weight (r = −0.68, *P*< 0.001).

G-DCA concentrations were significantly elevated in the ICP group compared with controls across the first, second, and third trimesters (21.36 vs. 48.37 ng/mL, 54.17 vs. 112.57 ng/mL, and 47.55 vs. 138.66 ng/mL, respectively) (*P* < 0.0001) (Fig. 4A–C). G-DCA demonstrated good diagnostic performance across trimesters (AUC 0.805–0.906, 95% CI 0.7142–0.9670) (Fig. 4D), with corresponding sensitivity, specificity, and optimal cut-off values summarized in Table 6. During the third trimester, G-DCA showed a positive correlation with TBA (r = 0.87, *P*< 0.001), whereas its correlations with gestational age at delivery (r = −0.67, *P*< 0.001) and neonatal birth weight (r = −0.68, *P*< 0.001) were negative (Fig. 4E–G). Detailed clinical characteristics and serum G-DCA level data are provided in Supplemental Material S2.

**Table 6:** Diagnostic performance of the G-DCA TRF-ICTS across trimesters

Trimester	AUC (95% CI)	Sensitivity (%)	Specificity (%)	Cut-off Value (ng/ml)
First trimester	0.805 (0.714–0.895)	78.0	80.0	29.6
Second trimester	0.906 (0.844–0.967)	92.0	78.6	68.3
Third trimester	0.864 (0.794–0.935)	90.2	81.5	68.1

ROC analysis was performed to evaluate diagnostic performance across trimesters. AUC values with 95% CI, sensitivity, specificity, and cut-off values are presented. Cut-off values were determined using the Youden index.

### Clinical utility of serum G-DCA in ICP

**Fig. 5:**
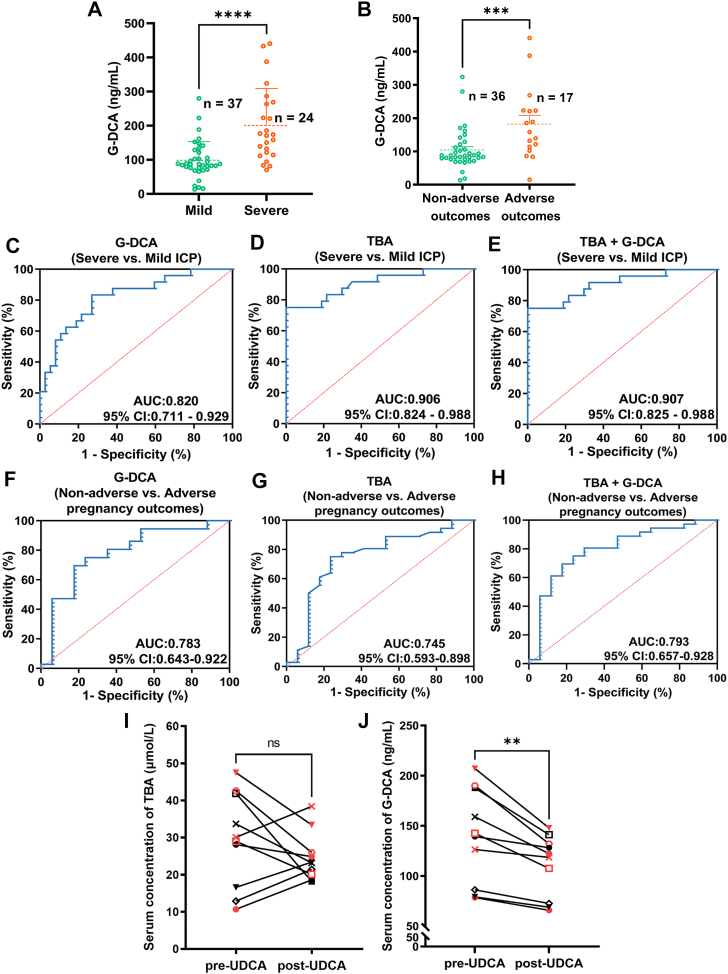
Diagnostic and clinical utility of serum G-DCA in ICP severity, pregnancy outcomes, and response to UDCA therapy. A: Serum G-DCA levels in mild (n = 37) and severe (n = 24) ICP patients. Significantly higher levels were observed in severe cases (*P*< 0.0001). B: Comparison of serum G-DCA levels between ICP patients with non-adverse (n = 36) and adverse pregnancy outcomes (n = 17). C–E: ROC curve analyses for distinguishing severe from mild ICP using G-DCA, TBA, and their combination, with AUC values of 0.820, 0.906, and 0.907, respectively. F–H: ROC curve analyses for predicting adverse pregnancy outcomes using G-DCA, TBA, and their combination, with AUC values of 0.783, 0.745, and 0.793, respectively. I–J: Changes in serum TBA and G-DCA levels before and after UDCA treatment (n = 10), with paired serum samples from each patient analyzed in triplicate for G-DCA.

As shown in Figure 5A, B, serum G-DCA levels were elevated in patients with severe ICP (*P*< 0.0001) and in those with adverse pregnancy outcomes (*P*< 0.001). ROC analysis showed that G-DCA yielded an AUC of 0.820 (95% CI: 0.711–0.929) for distinguishing severe from mild ICP, which was slightly lower than that of TBA (AUC = 0.906, 95% CI: 0.824–0.988). The combination of G-DCA and TBA slightly improved the performance (AUC = 0.907, 95% CI: 0.825–0.988) (Fig. 5C–E). For predicting adverse pregnancy outcomes, G-DCA achieved an AUC of 0.783 (95% CI: 0.643–0.922), slightly outperforming TBA (AUC = 0.745, 95% CI: 0.593–0.898). The combination of G-DCA and TBA showed a modest improvement (AUC = 0.793, 95% CI: 0.657–0.928) (Fig. 5F–H). As shown in Figure 5I, J, serum G-DCA levels decreased to varying degrees after UDCA treatment, whereas TBA levels showed inconsistent changes.

## Discussion

Traditionally, TBA have been regarded as the primary diagnostic marker for ICP. However, TBA reflects the total circulating bile acid pool and may also be elevated in certain hepatobiliary disorders (24). In addition, changes in TBA are often not pronounced in early-stage ICP, which may limit its diagnostic sensitivity and specificity (25). With the advancement of bile acid profiling technologies, increasing evidence suggests that alterations in specific bile acid species may provide additional insights into the pathophysiological characteristics of ICP and may serve as complementary indicators to TBA for improving diagnosis and disease stratification (26). G-DCA is a glycine-conjugated derivative of microbiota-generated DCA, formed in hepatocytes and involved in the enterohepatic circulation. Notably, although cholestasis may reduce the overall formation of secondary bile acids due to decreased bile flow into the intestine, circulating G-DCA levels may still increase in patients with ICP. This paradoxical elevation may be associated with impaired hepatocellular transport and disrupted cellular polarity, which could promote the spillover of conjugated bile acids into the systemic circulation, while enhanced glycine conjugation may further facilitate their solubility and release as part of a detoxification response (27, 28). This work sought to establish a TRFIA-based nanomicrosphere immunochromatographic strip capable of quantitatively measuring the small-molecule metabolite G-DCA in serum.

Immunochromatographic test strips commonly employ either the double-antibody sandwich or competitive format (29). The sandwich assay is primarily used for macromolecular antigens such as proteins, whereas the competitive format is more suitable for small molecules. To date, relatively few studies have applied this technique to the detection of the glycine-conjugated secondary bile acid G-DCA in serum. During TRF-ICTS development, Eu^3+^-FM exhibited a tendency to aggregate under high-concentration or high-electrolyte conditions, potentially compromising fluorescence intensity and assay stability (30). This issue was addressed using a two-step EDC/NHS-mediated conjugation strategy with intermediate washing, which improved conjugation efficiency and reduced nonspecific aggregation (31). Following systematic optimization of key assay parameters, including MES buffer pH, coating antigen concentration, labeled antibody amount, and reaction time, the assay demonstrated a suitable dynamic range (0–10 ng/ml) and high sensitivity (LOD: 0.074 ng/ml). The test strip also exhibited stable performance over 2 weeks under both 4°C and accelerated (37°C) conditions, although further studies are required to validate long-term stability. Overall, the assay showed satisfactory precision, accuracy and recovery, together with good specificity and resistance to interference, and demonstrated strong correlation and consistency with LC-MS/MS detection, indicating its potential for clinical application.

To further evaluate the clinical applicability of the developed G-DCA TRF-ICTS, serum G-DCA levels were measured across the first trimester (≤13^+6^weeks), second trimester (14–27^+6^weeks), and third trimester (≥28 weeks) in patients with ICP and healthy pregnant women, with consistently higher concentrations observed in the ICP group. G-DCA showed good diagnostic performance across all trimesters, with AUC values exceeding 0.8. The diagnostic cut-off values derived from ROC analysis were 68.3 ng/ml in the second trimester and 68.1 ng/ml in the third trimester. In contrast, a lower threshold of 29.6 ng/ml in the first trimester provided better discrimination between ICP and healthy pregnancies, highlighting the potential of G-DCA to improve early diagnosis of ICP, which may not be fully captured by conventional markers such as TBA. Furthermore, serum G-DCA levels were negatively correlated with gestational age at delivery and newborn birth weight, suggesting that elevated G-DCA levels may be associated with adverse pregnancy outcomes.

Notably, in ICP severity stratification, patients with severe disease (TBA ≥ 40 μmol/L) are at increased risk of adverse pregnancy outcomes. Third-trimester patients from the same cohort were stratified according to the 2024 clinical guidelines into mild and severe groups, as well as groups with and without adverse pregnancy outcomes (20). Serum G-DCA levels were elevated in patients with severe ICP and in those with adverse pregnancy outcomes. Subsequent ROC analysis showed that G-DCA achieved performance comparable to TBA in distinguishing severe from mild ICP and slightly better performance in predicting adverse pregnancy outcomes, while their combination provided modest improvements in AUC values. To evaluate the impact of UDCA treatment on G-DCA levels, serum G-DCA concentrations were measured in 10 patients with ICP before and after therapy, and changes in TBA levels were also recorded. G-DCA concentrations decreased to varying extents in all patients after treatment, whereas TBA levels decreased in most cases (6/10) but increased in four patients. This discrepancy may be attributed to mechanisms reported in previous studies. UDCA is incorporated into the circulating bile acid pool and contributes directly to TBA levels. Moreover, UDCA treatment alters bile acid composition, and reductions in endogenous bile acids may be offset by increased UDCA levels. In addition, in patients with relatively low baseline TBA levels, treatment-related changes may be less apparent (32, 33). In contrast, G-DCA levels decreased more consistently after treatment, suggesting that G-DCA may provide a more stable indicator for monitoring therapeutic response in ICP. Building on these findings, G-DCA may have potential as a complementary biomarker to TBA for early diagnosis, disease stratification, prediction of adverse pregnancy outcomes, and assessment of UDCA treatment response in ICP, thereby supporting more individualized management of patients.

In this study, a quantitative immunochromatographic test strip for serum G-DCA detection was developed, offering operational simplicity, rapid analysis, and potential clinical applicability. Differences in analytical methods, measurement systems, and reporting units may limit direct comparisons between biomarkers within the same disease context. This methodological limitation may be mitigated through appropriate calibration to enable more consistent interpretation (34). However, larger multicenter studies and additional paired pre- and post-UDCA treatment data are still needed to validate its diagnostic performance. The pathophysiological role of G-DCA in ICP also warrants further investigation.

## Data Availability

All data are contained within the manuscript and supplemental data.

## Supplemental Data

This article contains supplemental data.

## Conflict of Interest

The authors declare that they do not have any conflicts of interest with the content of this article.

## Supplemental Data

Supplemental Material 1 Refinement of the operational parameters for the G-DCA TRF-ICTS system.

Supplementary Material 2 Clinical information and G-DCA level data.

## References

[1] Dajti E., Tripodi V., Hu Y., Estiù M.C., Shan D., Mazzella G. (2025). Intrahepatic cholestasis of pregnancy. Nat. Rev. Dis. Primers..

[2] Liu Y., Wei Y., Chen X., Huang S., Gu Y., Yang Z. (2025). Genetic study of intrahepatic cholestasis of pregnancy in Chinese women unveils East Asian etiology linked to historic hbv epidemic. J. Hepatol..

[3] Jamshidi Kerachi A., Shahlaee M.A., Habibi P., Dehdari Ebrahimi N., Ala M., Sadeghi A. (2025). Global and regional incidence of intrahepatic cholestasis of pregnancy: a systematic review and meta-analysis. BMC Med..

[4] Tang M., Xiong L., Cai J., Fu J., Liu H., Ye Y. (2024). Intrahepatic cholestasis of pregnancy: insights into pathogenesis and advances in omics studies. Hepatol. Int..

[5] Ovadia C., Seed P.T., Sklavounos A., Geenes V., Di Ilio C., Chambers J. (2019). Association of adverse perinatal outcomes of intrahepatic cholestasis of pregnancy with biochemical markers: results of aggregate and individual patient data meta-analyses. Lancet.

[6] Zou S., Dong R., Wang J., Liang F., Zhu T., Zhao S. (2021). Use of data-independent acquisition mass spectrometry for comparative proteomics analyses of sera from pregnant women with intrahepatic cholestasis of pregnancy. J. Hepatol..

[7] Fuchs C.D., Simbrunner B., Baumgartner M., Campbell C., Reiberger T., Trauner M. (2025). Bile acid metabolism and signalling in liver disease. J. Hepatol..

[8] Wang L., Wang Z., Zhao Y., Yang B., Huang G., Li J. (2024). Gut microbiota-mediated bile acid metabolism aggravates biliary injury after liver transplantation through mitochondrial apoptosis. Int. Immunopharmacol..

[9] Dean A.E., Guzior D.V., Quinn R.A., Gaulke C.A., Anakk S. (2026). Serum cholic acid and cecal faecalibaculum increase in a male-specific manner in a murine hepatocellular carcinoma model. J. Lipid Res..

[10] Zhu B., Ma Z., Zhu Y., Fang L., Zhang H., Kong H. (2021). Reduced glycodeoxycholic acid levels are associated with negative clinical outcomes of gestational diabetes mellitus. J. Zhejiang Univ. Sci. B.

[11] Drzymała-Czyż S., Dziedzic K., Szwengiel A., Krzyżanowska-Jankowska P., Nowak J.K., Nowicka A. (2022). Serum bile acids in cystic fibrosis patients - glycodeoxycholic acid as a potential marker of liver disease. Dig. Liver Dis..

[12] Yang X., Qin H., Ling J., Dong L., Jiang Y., Zou S. (2026). Rapid quantification of bile acids in serum by LC-MS/MS and application to serum bile acid profile in voriconazole administered patients with invasive fungal infections. Bioanalysis.

[13] Roumain M., Muccioli G.G. (2025). Development and application of an lc-ms/ms method for the combined quantification of oxysterols and bile acids. J. Lipid Res..

[14] Thomas S.N., French D., Jannetto P.J., Rappold B.A., Clarke W.A. (2022). Liquid chromatography-tandem mass spectrometry for clinical diagnostics. Nat. Rev. Methods Primers..

[15] Feng R., Wang Y., Chen Y., Li T., Zhou H., Zhan Z. (2026). Development of a monoclonal antibody-based time-resolved fluorescence immunochromatographic assay strip for rapid and quantitative determination of s100b in serum. Anal. Bioanal. Chem..

[16] Lu Z., Niu J., Li Y., Li Y., Feng D., Xu S. (2025). Cascade signal amplification of time-resolved fluorescence immunoassay through enzyme catalysis coupled with dissolution enhancement. Anal. Chem..

[17] Shen T., Yang F., Wu J., Qin Y., Zhou X., Zhao X. (2025). Establishment of time-resolved fluorescence immunoassay for thyrotropin receptor antibodies and clinical application. J. Fluoresc..

[18] Huang H., Gao J., Dong R., Wang R., Li L., Wang G. (2025). Detection of serum lactate dehydrogenase a and its metabolites on placental function in patients with intrahepatic cholestasis of pregnancy. Int. Immunopharmacol.

[19] Liang Y.F., Yang J.Y., Shen Y.D., Xu Z.L., Wang H. (2025). A breakthrough of immunoassay format for Hapten: recent insights into noncompetitive immunoassays to detect small molecules. Crit. Rev. Food Sci. Nutr..

[20] Obstetrics Subgroup, S.o.O., Gynecology, C.M.A., and CMA (2024). Guidelines for clinical diagnosis, treatment and management of intrahepatic cholestasis of pregnancy. Zhonghua Fu Chan Ke Za Zhi..

[21] Shen Y., Liu K., Luo X., Guan Q., Cheng L. (2022). A simple and reliable bile acid assay in human serum by LC-MS/MS. J. Clin. Lab. Anal..

[22] Wang L., Sun J., Ye J., Wang L., Sun X. (2022). One-step extraction and simultaneous quantitative fluorescence immunochromatography strip for AFB1 and Cd detection in grain. Food Chem..

[23] Pedreira-Rincón J., Rivas L., Comenge J., Skouridou V., Camprubí-Ferrer D., Muñoz J. (2025). A comprehensive review of competitive lateral flow assays over the past decade. Lab. Chip.

[24] Yao L., Zhou J., Tan Z., Li C., He T., Yin Y. (2025). Total bile acid concentrations and adverse perinatal outcomes in Chinese intrahepatic cholestasis of pregnancy including asymptomatic hypercholanemia of pregnancy: a retrospective cohort study. BMC Pregnancy Childbirth.

[25] Hobson S.R., Cohen E.R., Gandhi S., Jain V., Niles K.M., Roy-Lacroix M. (2024). Guideline no. 452: diagnosis and management of intrahepatic cholestasis of pregnancy. J. Obstet. Gynaecol. Can..

[26] Tang B., Tang L., Li S., Liu S., He J., Li P. (2023). Gut microbiota alters host bile acid metabolism to contribute to intrahepatic cholestasis of pregnancy. Nat. Commun..

[27] Fleishman J.S., Kumar S. (2024). Bile acid metabolism and signaling in health and disease: molecular mechanisms and therapeutic targets. Signal Transduct. Target Ther..

[28] Ma Z., Liu Y., Chai L., Jin G., Sun Y., Zhou S. (2023). Metabolic changes in bile acids with pregnancy progression and their correlation with perinatal complications in intrahepatic cholestasis of pregnant patients. Sci. Rep..

[29] Zvereva E.A., Hendrickson O.D., Dzantiev B.B., Zherdev A.V. (2024). Comparison of competitive and sandwich immunochromatographic analysis in the authentication of chicken in meat products. Anal. Biochem..

[30] Farka Z., Juřík T., Kovář D., Trnková L., Skládal P. (2017). Nanoparticle-based immunochemical biosensors and assays: recent advances and challenges. Chem. Rev..

[31] Sapsford K.E., Algar W.R., Berti L., Gemmill K.B., Casey B.J., Oh E. (2013). Functionalizing nanoparticles with biological molecules: developing chemistries that facilitate nanotechnology. Chem. Rev..

[32] Beuers U., Banales J.M., Karpen S.J., Keitel V., Williamson C., Trauner M. (2025). The history and future of bile acid therapies. J. Hepatol..

[33] Sinakos E., Marschall H.U., Kowdley K.V., Befeler A., Keach J., Lindor K. (2010). Bile acid changes after high-dose ursodeoxycholic acid treatment in primary sclerosing cholangitis: relation to disease progression. Hepatology.

[34] Plebani M. (2013). Harmonization in laboratory medicine: the complete picture. Clin. Chem. Lab. Med..

