# Supplementary material for: Development and application of a fluorescent immunochromatographic assay for metabolite G-DCA quantification in ICP
Source: J Lipid Res. 2026 Apr 16;67(5):101039. doi: 10.1016/j.jlr.2026.101039 (PMC13202283; doi:10.1016/j.jlr.2026.101039)
Supplement: Supplemental Material 1 — Refinement of the operational parameters for the G-DCA TRF-ICTS system. [file mmc1.docx]

***Optimization of the G-DCA TRF-ICTS***

***Optimization of coupling strategy*** Two coupling strategies were evaluated: the one-step method, in which EDC/NHS-activated Eu³⁺-FM were directly conjugated with antibodies without intermediate washing, and the two-step method, in which activated microspheres were washed prior to antibody conjugation. Samples spiked with 0.5 and 1.0 ng/mL G-DCA in Tris–HCl buffer were used as low-level positive samples, while Tris–HCl buffer alone served as the negative control. The fluorescence signal is expressed as the T/T₀ ratio, where T represents the fluorescence intensity of the test line for the sample and T₀ represents that of the negative control. The performance of each strategy was assessed based on the T/T₀ values. All experiments were performed in triplicate.

***Optimization of MES buffer pH*** The effect of MES buffer pH on the analytical performance of the G-DCA TRF-ICTS was evaluated at pH 5.0, 5.5, 6.0, 6.5, and 7.0. Fluorescent probes were prepared by dispersing Eu³⁺-FM in MES buffers at different pH values, followed by conjugation with equal amounts of G-DCA-mAb. The probe-containing buffer was mixed with positive samples (1.0 ng/mL G-DCA) or negative control (Tris–HCl buffer), and the resulting mixtures were applied to the test strips for analysis. The performance under each pH condition was assessed based on the T/T₀ values. All experiments were performed in triplicate.

***Optimization of G-DCA-OVA coating concentration*** To determine the optimal coating concentration of G-DCA-OVA on the T line, five concentrations (0.5, 1.0, 1.5, 2.0, and 2.5 mg/mL) were evaluated. The C line was coated with goat anti-mouse IgG (1.0 mg/mL), and reagents were dispensed onto the nitrocellulose membrane at 1.0 μL/cm. TRF-ICTS from the same batch were used to measure the fluorescence signal using a positive sample (1.0 ng/mL G-DCA) and a negative control (Tris–HCl buffer). The fluorescence intensity of the T line and the corresponding T/T₀ values were analyzed to determine the optimal coating concentration.

***Optimization of reaction time*** To determine the optimal reaction time of the assay, samples spiked with 1.0 ng/mL G-DCA in Tris–HCl buffer were used as positive samples, while Tris–HCl buffer alone served as the negative control. Fluorescence signals of the T line were recorded at 2 min intervals over a 20 min period using an immunoquantitative reader, and expressed as the T/T₀ ratio, where T represents the fluorescence intensity of the sample and T₀ represents that of the negative control. The time point at which the T line signal reached a stable plateau was defined as the optimal reaction time. All experiments were performed in triplicate.
